# Histopathological Changes in the Kidney following Congestive Heart Failure by Volume Overload in Rats

**DOI:** 10.1155/2017/6894040

**Published:** 2017-07-31

**Authors:** Noureddin B. Aboryag, Doaa M. Mohamed, Lukas Dehe, Mohammed Shaqura, Sacha Treskatsch, Mehdi Shakibaei, Michael Schäfer, Shaaban A. Mousa

**Affiliations:** ^1^Department of Anaesthesiology and Intensive Care Medicine, Charité University Berlin, Campus Virchow Klinikum and Campus Charité Mitte, Berlin, Germany; ^2^Department of Anatomy, Ludwig-Maximilians-University Munich, Munich, Germany

## Abstract

**Background:**

This study investigated histopathological changes and apoptotic factors that may be involved in the renal damage caused by congestive heart failure in a rat model of infrarenal aortocaval fistula (ACF).

**Methods:**

Heart failure was induced using a modified approach of ACF in male Wistar rats. Sham-operated controls and ACF rats were characterized by their morphometric and hemodynamic parameters and investigated for their histopathological, ultrastructural, and apoptotic factor changes in the kidney.

**Results:**

ACF-induced heart failure is associated with histopathological signs of congestion and glomerular and tubular atrophy, as well as nuclear and cellular degeneration in the kidney. In parallel, overexpression of proapoptotic Bax protein, release of cytochrome C from the outer mitochondrial membrane into cell cytoplasm, and nuclear transfer of activated caspase 3 indicate apoptotic events. This was confirmed by electron microscopic findings of apoptotic signs in the kidney such as swollen mitochondria and degenerated nuclei in renal tubular cells.

**Conclusions:**

This study provides morphological evidence of renal injury during heart failure which may be due to caspase-mediated apoptosis via overexpression of proapoptotic Bax protein, subsequent mitochondrial cytochrome C release, and final nuclear transfer of activated caspase 3, supporting the notion of a cardiorenal syndrome.

## 1. Introduction

Heart failure, a progressive disease marked by repeated hospitalizations for episodes of acute decompensation, is frequently complicated by kidney dysfunction—one of the most important risk factors for poor clinical outcome and death [1]. In congestive heart failure, the heart cannot deliver oxygen at a rate proportionate to the demands of the metabolizing tissues that may result in damage to other organ systems such as the kidney [2–4]. It is well established that heart performance and kidney function are closely interconnected and dysfunction of one organ often leads to a deterioration of the function of another which is known as cardiorenal syndrome [5]. Consistently, more than 1 million patients present to hospitals in the United States with acutely decompensated heart failure every year and approximately one-third of these patients develop kidney injury [6]. Consequently, these patients who develop kidney dysfunction after heart failure have a significantly higher mortality rate [7, 8]. In addition, there is emerging evidence that heart failure can also be considered as an inflammatory state that contributes to gradual toxic injury to renal cells including apoptosis which may lead to chronic kidney damage and functional loss [5, 9, 10]. Recently, Cho et al. showed that the number of TUNEL-positive apoptotic tubular cells significantly increased in the kidneys of rats with myocardial infarction [11].

Once cells receive the apoptotic stimulus, they constitute specific pathways, including the disruption of mitochondrial transmembrane potential, followed by the release of mitochondrial proteins like cytochrome C and the activation of caspase subtypes within the apoptosome complex leading to cell death [12–14]. Recent evidence is emerging that the mitochondria-mediated apoptosis is initiated by a variety of apoptosis-inducing signals that cause the imbalance of the major apoptosis regulator such as Bcl-2 and Bax [15]. Therefore, the aim of our current study was to investigate the histopathological and ultrastructural changes in the kidney in a modified experimental rat model of infrarenal aortocaval fistula-induced heart failure. Moreover, we investigated possible alterations in the expression of apoptotic factors such as bax protein, cytochrome C, and caspase-3 as well as activated caspase-3.

## 2. Materials and Methods

### 2.1. Animals

Male Wistar rats, 280–300 g (Harlan Winkelmann, Borchen, Germany), were maintained on standard laboratory rat chow and water ad libitum. The animals were kept on a 12 h light–dark cycle with a temperature of 23°C and a humidity of 75%. This study was carried out in accordance with the European directive introducing new animal welfare and care guidelines (2010/63/EU). IRB approval for animal experiments was obtained from local authorities (Landesamt für Gesundheit und Soziales, Berlin, Germany). Surgical procedures were performed under isoflurane (ACF induction) and tiletamine/zolazepam (hemodynamic measurements) anesthesia, and all efforts were made to minimize suffering. Postsurgical analgesia was provided by metamizole (40 mg/kg s.c.).

### 2.2. Experimental Heart Failure Model

The needle technique to induce an infrarenal aortocaval fistula (ACF) has previously been described by Garcia and Diebold using an 18G needle [16, 17]. In a modified approach, a laparotomy was performed and the aorta was punctured by using a 16G needle (Braun, Melsungen, Germany) distal to the renal arteries [17]. Then, the needle was advanced through the aortic wall into the adjacent inferior vena cava. After temporarily compressing the aorta and venous vessels above and below the puncture site, the needle was carefully withdrawn and the aortic puncture site sealed with a drop of cyanoacrylate glue. Patency of the fistula (*n* = 5 rats for morphometric/hemodynamic measurements, for immunohistochemistry and for electron microscopy, resp.) was visualized by the pulsatile flow of oxygenated blood into the vena cava inferior from the infrarenal aorta [17, 18]. Sham-operated rats (*n* = 5 rats for morphometric/hemodynamic measurements, for immunohistochemistry and for electron microscopy, resp.) also received a laparotomy with the vessels temporarily compressed, but without any puncture of the aorta.

### 2.3. Morphometric Data

After 28 ± 2 days of fistula induction, the animals were sacrificed in isoflurane anesthesia and blood, heart, lung, and kidney tissues were quickly removed. The wet weight of heart, lung, and kidney tissues (*n* = 5 rats) was measured by a balance and normalized to the body weight of the individual animal to obtain the respective indices.

### 2.4. Hemodynamic Measurements

For hemodynamic measurements, the “closed chest” method in spontaneously breathing rats was used as described previously [17, 19]. All measurements were performed under tiletamine/zolazepam anesthesia (Zoletil®, 10 mg/kg s.c. followed by 50 mg/kg i.m.) 28 ± 2 days after fistula induction [17, 20]. Measurements were registered and analyzed by the PowerLab® system/software (AD Instruments, New Zealand). After tracheotomy, a PE-50-tubing catheter was inserted via the left jugular vein into the superior vena cava for the assessment of central venous pressure. Arterial blood pressure was measured by cannulating the right carotid artery with a microtip pressure-volume conductance catheter (Millar®, SPR-838 NR). Intraventricular pressures and volumes were registered by further advancing the catheter into the left ventricle and optimizing its position aiming for maximal stroke volume (SV). For measurement of the parallel conductance volume, 100 *μ*l of 15% saline was injected into the central venous line as a correction factor for the blood-left ventricle (LV) tissue interface. Heart rate was derived from the ECG signal. After completion of the hemodynamic measurements, animals (*n* = 5 rats per group) were killed by exsanguination and organs were eviscerated for further determinations.

### 2.5. Tissue Preparation

Rats were deeply anesthetized with isoflurane and perfused transcardially with 100 ml 0.1 m PBS (pH 7.4) and 300 ml cold PBS containing 4% paraformaldehyde and 0.2% picric acid (pH 7.4; fixative solution) for light and fluorescence microscopy and with 4% paraformaldehyde/0.1% glutaraldehyde/0.2% picric acid solution (pH 7.4) for electron microscopy, respectively [21]. The kidneys were removed, renal tissue postfixed for 90 min at 4°C in the fixative solution, and cryoprotected overnight at 4°C in PBS containing 10% sucrose. Consecutive sections (6 *μ*m thick) prepared on cryostat were mounted onto gelatin-coated slides.

### 2.6. Histological Examination

Kidney tissue sections from 5 rats were stained with hematoxylin and eosin as previously described [22]. For histological analysis, random nonoverlapping fields from the cortex-to-corticomedullary region of each kidney section were captured using a 40x magnification lens by light microscopy (Zeiss Axioplan photomicroscope equipped with a digital camera).

### 2.7. Transmission Electron Microscopy

Tissue of the kidney from 5 rats was processed for electron microscopy as described previously [21, 23]. Small pieces of tissue were postfixed in 1% tannic acid (in 0.1 m PBS) and 1% osmium tetroxide solution (in 0.1 m PBS), dehydrated in ethanol, and embedded in Epon. Semithin and ultrathin sections were cut on a Reichert Ultracut (Leica, Germany), followed by contrasting with 2% uranyl acetate/lead citrate. Finally, ultrathin sections were examined under a transmission electron microscope (TEM 10, Zeiss, Germany). Semithin sections were stained 1 to 2 minutes in 1% Toluidine Blue (Merck, Darmstadt, Germany), rinsed several times in purified water, and examined under a light microscope (Axiophot 100; Zeiss, Germany).

## 3. Apoptosis Assay

The assessment of liver apoptosis was performed in situ using terminal deoxynucleotidyl transferase-mediated dUTP nick end-labeling (TUNEL) assays (Chemicon Apo-Direct Tunel Assay Kit; Merck Millipore, Darmstadt, Germany) for the detection of the internucleosomal DNA fragmentation, characteristic for apoptosis according to the manufacturer's instructions. Briefly, 6 *μ*m sections of paraformaldehyde-fixed liver tissue were postfixed with precooled fixative (ethanol/acetic acid) for 5 min at −20°C. After PBS wash, the sections were then immersed in 1x TdT equilibration buffer at room temperature for 30 min followed by incubation with working strength TdT enzyme for 1 h at 37°C. The reaction was terminated with working strength stop/wash buffer, and the sections were washed with PBS. Then, the sections were incubated with FITC conjugated anti-digoxigenin at room temp for 30 min. After PBS washing, the nuclei were stained bright blue with 4′-6-diamidino-2-phenylindole (DAPI) (0.1 *μ*g/ml in PBS) (Sigma). As a negative control, sections were incubated in the absence of TdT enzyme.

### 3.1. Double Immunofluorescence Staining

Double immunofluorescence staining of cytochrome C in the kidney was performed as described previously [24, 25]. Kidney sections were incubated overnight with the following primary antibodies: (1) monoclonal mouse anti-mitochondria (catalogue number MA5-12017, Thermo Fisher Scientific, Rockford, IL) in combination with rabbit polyclonal anti-cytochrome C (catalogue number 4280, Cell Signalling, Danvers, MA); (2) rabbit polyclonal anti-cleaved caspase-3 (catalogue number 9661, Cell Signalling), anti-caspase-3 (catalogue number ab13847, Abcam; Cambridge, MA), and anti-Bax protein (catalogue number SC-526, Santa Cruz Biotechnology, Texas). After incubation with primary antibodies, the tissue sections were washed with PBS and then incubated with red fluorescent Alexa Fluor 594 donkey anti-rabbit antibody (Vector Laboratories) in combination with green fluorescent Alexa Fluor 488 goat anti-mouse antibody (Invitrogen, Germany). Thereafter, sections were washed with PBS, and the nuclei stained bright blue with 4′-6-diamidino-2-phenylindole (DAPI) (0.1 *μ*g/ml in PBS) (Sigma).

### 3.2. Quantification of Immunostaining

The method of quantification of colocalization of mitochondria with cytochrome C in the kidney has been described in detail elsewhere [25, 26]. Briefly, quantification of immunofluorescent colocalization of mitochondrial marker with cytochrome C in kidney tissue sections was performed by using the Zeiss Zen 2009 software Carl Zeiss Micro-Imaging GmbH (Göttingen, Germany). Colocalization of proteins of interest was quantified by calculating the colocalization coefficient as derived from Mander's article based on Pearson's correlation coefficient [26]. Images were adjusted to a threshold to exclude background fluorescence and gated to include intensity measurements only from positively stained cells. The number of caspase-3-stained nuclei was determined by the formula: caspase-3-stained nuclei/total number of DAPI-stained nuclei × 100. For image analysis, using the area of the whole stained tissue section, five rats per group were used for analysis. Data were expressed as means ± SEM.

### 3.3. Statistical Analysis

All tests were performed using SPSS 20.0 software program. Normal distribution was analyzed with the Kolmogorov-Smirnov test. The results are expressed as means ± SEM. Statistical significance between the two groups was analyzed by the Student *t*-test or Mann–Whitney test as appropriate. *p* < 0.05 was considered statistically significant. All tests were performed using Sigma Plot 13.0 statistical software.

## 4. Results

### 4.1. Increased Heart and Lung Weight Indices following Congestive Heart Failure

**Figure 1:**
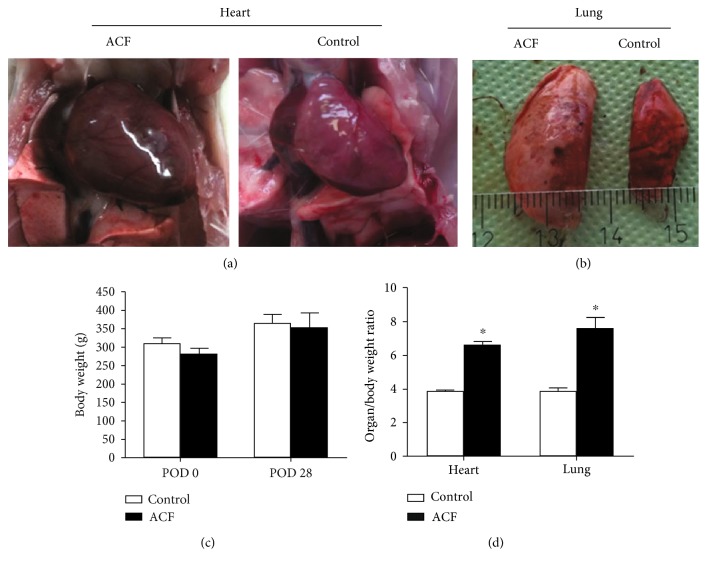
(a) and (b) show the enlarged size of the heart (a) and lung (b) in rats with ACF-induced heart failure compared to sham-operated controls. These animals revealed significantly increased heart and lung weight indices (d), but not body weight. (c)∗ denotes significant differences compared to control (*p* < 0.01, Student *t*-test).

Body weights of rats with ACF-induced heart failure were not significantly different from sham-operated controls (Figure 1). In contrast, the heart and lung weight indices were significantly increased at 28 ± 2 days after ACF-induced heart failure (*p* < 0.01) (Figure 1).

### 4.2. Systolic and Diastolic Dysfunction in ACF Rats

In vivo hemodynamic measurements of control and ACF rats showed that central venous (CVP) and left end-diastolic pressure (LVEDP) were significantly increased in ACF rats (*p* < 0.01) (Table 1). While the left ventricular end-diastolic and end-systolic volumes (LVEDV and LVESV, resp.) were significantly elevated (*p* < 0.01), the left ventricular ejection fraction (LVEF) (*p* < 0.01), and the maximum rate of pressure development (*p* < 0.01) were significantly reduced (Table 1).

### 4.3. Histopathological Changes in the Kidney following Congestive Heart Failure

**Figure 2:**
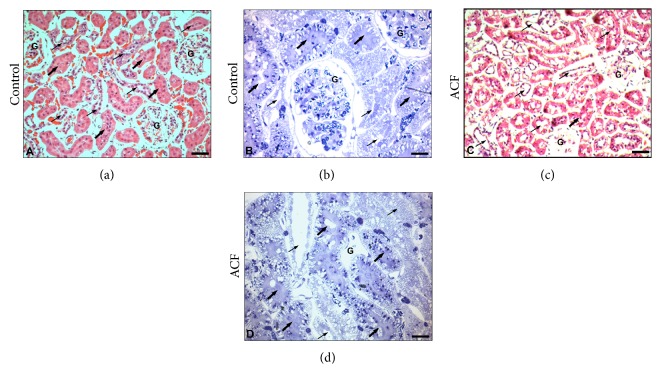
Light microscopic photographs of representative kidney sections of control and ACF adult rats. (a) and (b) show control haematoxylin-eosin- (a) and toluidine blue-stained (b) rat kidney with normal distal (thin arrow), proximal (thick arrow) tubular and glomerulus (G) structures are seen in the medulla of control kidney. (c) and (d) show ACF haematoxylin-eosin- (c) and toluidine blue-stained (d) rat kidney with histopathological changes in atrophic glomeruli (G) with atrophic changes such as widening of Bowman's space with haemolysis, necrosis with obvious degeneration in nuclei, and apoptotic cell death. Also, histopathological changes in atrophic distal (thin arrow) and proximal (thick arrow) tubular structures are shown with epithelial shedding and loss of brush border of proximal tubules as well as desquamation of tubular epithelium of distal tubules in the medulla. (a), (c): ×120; (b), (d): ×200.

In kidney tissue sections of control rats, a normal structure of glomeruli as well as proximal and distal tubuli was observed (Figures 2(a) and 2(b)). In contrast, kidney sections of ACF rats showed signs of morphological and pathological changes (Figures 2(c) and 2(d)). In the glomerular region of the kidney, several glomeruli showed atrophic changes, widening of Bowman's space with obvious degeneration of cells and losing the prominent glomerular structure suggesting apoptotic cell death. As compared to controls (Figures 2(a) and 2(b)), the proximal convoluted tubuli in ACF animals showed atrophic changes including epithelial shedding and loss of brush border (Figures 2(c) and 2(d)). Furthermore, pyknotic nuclei and desquamated cells (Figure 2) were seen in the tubular region. Tubular lumen was found to be sometimes obliterated due to degeneration and swelling of lining epithelial cells. The distal kidney tubuli of ACF rats revealed signs of degeneration, manifested by desquamation of epithelial tubular cells (Figure 2).

## 5. Detection of DNA Fragmentation by Tunel Staining in Kidney Cells following Congestive Heart Failure

**Figure 3:**
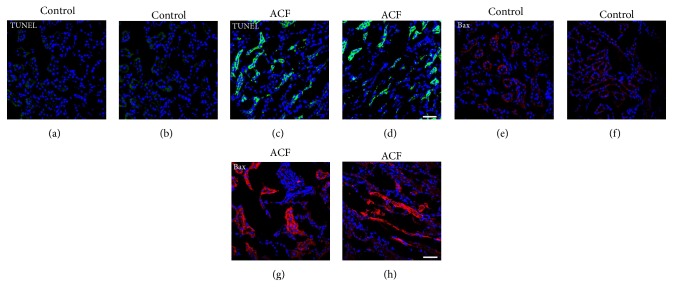
Confocal microscopy of TUNEL staining ((a), (b), (c), and (d)) and proapoptotic Bax ((e), (f), (g), and (h)) in the kidney of control and ACF adult rats. (a), (b), (c), and (d) showed TUNEL-positive (green fluorescence) with DAPI-counterstained nucleus (blue fluorescence) immunofluorescence of the kidney in control or ACF adult rats. Note that apoptotic tubular cells were detected in the kidney following ACF-induced heart failure in rats (c) and (d); however, no staining was found in controls (a) and (b). (e), (f), (g), and (h) Confocal microscopy of proapoptotic Bax protein (red fluorescence) with DAPI-counterstained nucleus (blue fluorescence) immunofluorescence in the kidney in control or in ACF adult rats. Note that the absence or weak Bax immunostaining was detected in the renal tubular cell cytoplasm of control kidney (e) and (f). However, strong positive immunofluorescence staining within renal tubular cell cytoplasm after heart failure induction was detected (g) and (h). Bar = 20 *μ*m.

Apoptosis was confirmed by TUNEL staining. In ACF kidneys, apoptosis was observed by TUNEL staining predominantly in tubular epithelial cells. Both proximal and distal tubule cells displayed DAPI-positive nuclei that were intensely TUNEL positive (Figures 3(b) and 3(f)). Furthermore, TUNEL-positive cells were not present in controls (Figures 3(a) and 3(e)).

### 5.1. Expression of Proapoptotic Bax in Kidney Cells following Congestive Heart Failure

To corroborate the TUNEL findings and to investigate the possible mechanisms mediating kidney apoptosis, we measured apoptotic factors in the kidney following ACF-induced heart failure. Examining kidney tissue of control rats by immunofluorescence confocal microscopy, proapoptotic Bax immunostaining was faintly observed in the proximal and distal tubuli; however, no staining was found in the glomeruli or interstitium (Figures 3(c) and 3(g)). After the induction of ACF-induced heart failure, overt Bax staining was noticeable in the damaged proximal and distal tubular cells of kidney tissue sections (Figures 3(d) and 3(h)). Also, some glomerular cells showed positive staining (Figures 3(d) and 3(h)).

### 5.2. Mitochondrial Leakage of Cytochrome C into the Cytosol of Kidney Cells following Congestive Heart Failure

**Figure 4:**
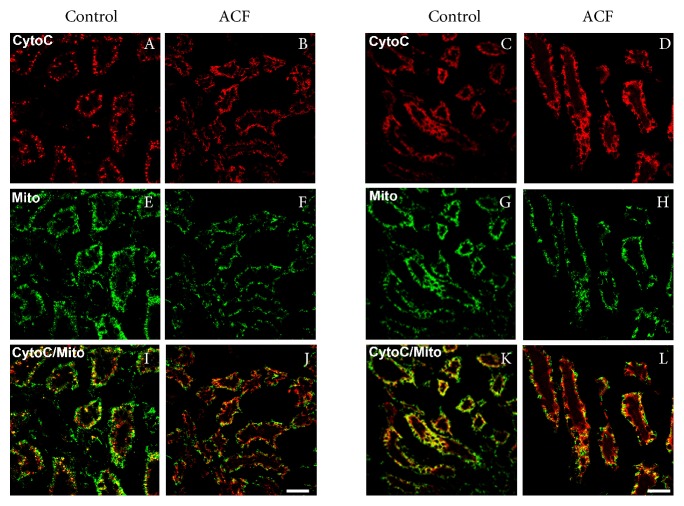
Confocal microscopy of cytochrome C (red fluorescence) with mitochondrial marker (green fluorescence) double immunofluorescence of the renal tubular cells in the kidney of control and ACF adult rats. Note that the cytochrome C immunostaining overlapped with the mitochondrial marker in the cytoplasm of renal tubular cells of control kidney (A, E, I and C, G, K). However, cytochrome C immunofluorescence was confined primarily within the cytoplasm after heart failure induction, where it no longer colocalized with mitochondria within renal tubular cells (B, E, J and D, H, L). Bar = 20 *μ*m.

**Figure 5:**
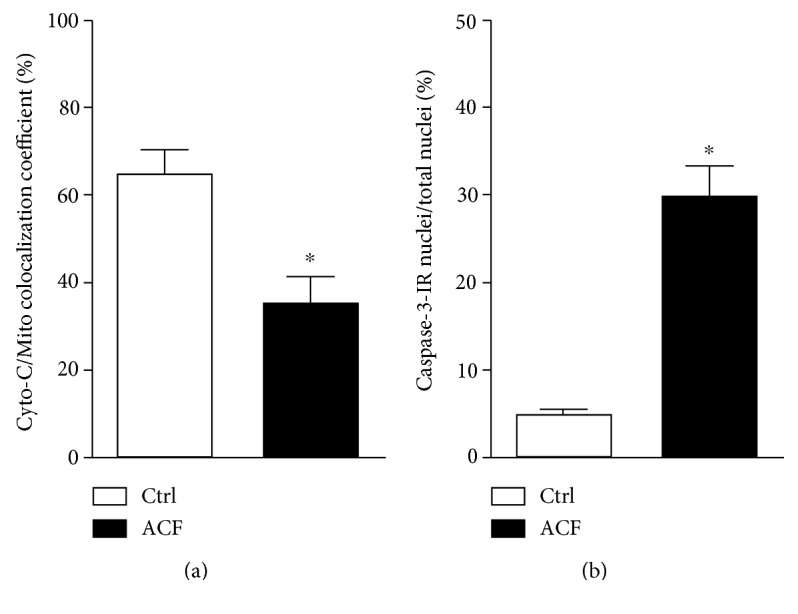
(a) Quantitative analysis of immunofluorescence microscopy of mitochondrial leakage of cytochrome C into the cytosol as well as nuclear transfer of caspase-3 as activated caspase-3 into the nucleus of kidney cells following congestive heart failure. Quantitative analysis of immunofluorescence microscopy of the colocalization coefficient of cytochrome C and mitochondrial marker showing a significant reduction of their colocalization in the liver in ACF animals compared to in controls (*n* = 5, *p* < 0.05, Student *t*-test). (b) Quantitative analysis of immunofluorescence microscopy for cleaved caspase-3-IR renal tubular cell nuclei in the kidney of ACF animals relative to the control (∗ denotes significant differences compared to control, *n* = 5, *p* < 0.05, Student *t*-test).

In kidney tissue sections of control rats, cytochrome C immunoreactivity was localized primarily to mitochondria as indicated by an almost complete overlap with the mitochondrial marker within renal tubular cells (Figure 4()). In contrast, cytochrome C immunofluorescence was found primarily within the cytoplasm especially in perinuclear area of renal tubular cells after heart failure induction, where it no longer colocalized with mitochondrial marker (Figure 4()). Quantification of the colocalization coefficient of cytochrome C and mitochondrial marker within kidney sections showed a significant reduction of their colocalization in ACF animals compared to controls (Figure 5(a)) indicating that cytochrome C had leaked from mitochondria into the cytoplasm.

### 5.3. Nuclear Transfer of Caspase-3 as Activated Caspase-3 into the Nucleus of Kidney Cells following Congestive Heart Failure

**Figure 6:**
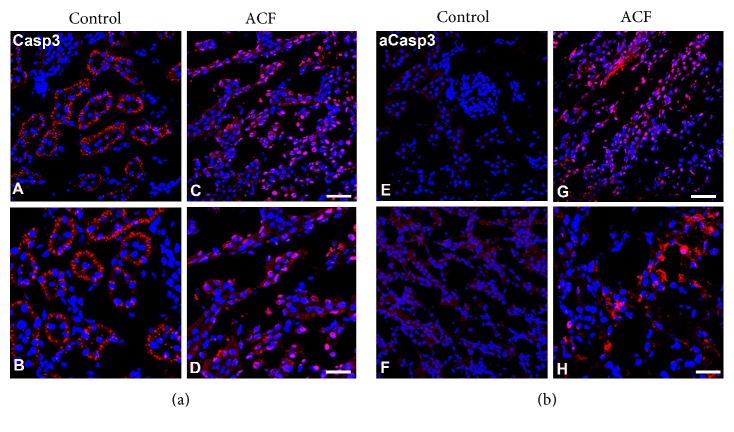
Confocal immunofluorescence microscopy of caspase-3 (red fluorescence) using an antibody detecting pro-caspase-3 recombinant protein (A–D) or cleaved caspase-3-recombinant protein (E–H) with DAPI-counterstained nuclei (blue fluorescence) in the kidney sections of control or ACF adult rats. (A–D) Note that caspase-3 immunoreactivity was confined primarily to well-defined subcellular organelles like structures in renal tubular cells within the kidney in control rats. In contrast in ACF rats, caspase-3 immunofluorescence was transferred into the perinuclear area of cells or inside the nuclei of renal tubular cells (C and D) indicating an activation of proapoptotic factor caspase-3. (E–H) Confocal immunofluorescence microscopy caspase-3 (red fluorescence) and DAPI-counterstained nuclei (blue fluorescence) in kidney sections using an antibody detecting exclusively cleaved caspase-3-recombinant protein. (G and H) showed that cleaved caspase-3 immunoreactivity was confined primarily to perinuclear area of cells or nuclei within renal tubular cells of ACF rat kidney (D); however, no staining was found in the control (F). Bar = 20 *μ*m.

We evaluated the localization of caspase-3 using antibodies that detect cleaved caspase-3-recombinant protein alone or with pro-caspase-3-recombinant proteins in kidney sections by confocal immunofluorescence microscopy in ACF and sham-operated control rats (Figure 6). Immunofluorescence confocal microscopy of kidney sections of control rats revealed that caspase-3 immunoreactivity was confined primarily to well-defined subcellular structures in proximal and distal tubular cells of the kidney, no staining was found in the glomeruli or interstitium. In contrast, caspase-3 immunofluorescence was translocated into perinuclear area of cells or nuclei within tubular cells in ACF rats. Also, some glomerular cells show positive staining (Figure 6) indicating that caspase-3 translocated in the active form. Therefore, we used the apoptotic marker cleaved activated caspase-3 which is absent under normal conditions and detectable only during cell apoptosis. Indeed, using an antibody which detects cleaved caspase-3-recombinant protein in kidney sections of ACF rats revealed that cleaved caspase-3 immunoreactivity was confined primarily to perinuclear area of cells or nuclei within tubular cells (Figure 6(b)).

Importantly, the number of caspase-3-IR nuclei as well as cleaved caspase-3-IR nuclei cells in relation to the total number of DAPI-stained nuclei was significantly higher in ACF rats than in controls (*p* < 0.05) (Figure 6(b)).

### 5.4. Transmission Electron Microscopy Evaluation of Kidney Cells following Congestive Heart Failure

**Figure 7:**
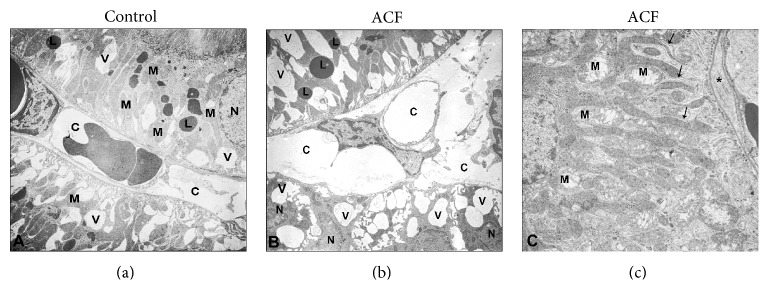
Transmission electron micrograph of the rat kidney in control (a) and ACF (b and c) groups. (a) shows normal ultrastructure of the control kidney. Note; untreated control of kidney cells consist of vital nucleus (N), well-developed cell organelles like mitochondria (M), lysosome (L), and a well-organized cytoplasm. (b) Electron microscopic demonstration of the ACF kidney tissue. Note that ultrastructure evaluations show a marked cytoplasmic vacuolization (V) of cells and capillary (C) dilation in the peritubular area and dilated cell organelles and cell nuclei (N) became condensed indicating intrinsic apoptotic events. (c) Many swollen mitochondria situated between the extensive inholdings (arrow) of the basolateral plasma membrane (∗) that create the lateral cell processes of distal tubules become dilated and exhibit a crista disarrangement and partial cristolysis. ×5000; bars:1 *μ*m.

Ultrastructural examination of kidney from control rats contrasted with uranyl acetate and lead citrate presents normal tissue of kidney ultrastructure containing mitochondria and endoplasmic reticulum (Figure 7(a)). In contrast, kidney sections from ACF rats present a marked cytoplasmic vacuolization of cells and dilated endoplasmic reticulum, mitochondria, and pleomorphic lysosomes containing flocculent material. In addition, the changes in the structure of renal glomeruli and tubular cells of ACF kidney included signs of matrix vacuolization, dilation of capillary, and degeneration of cell nuclei. In edematous regions, the space between individual tubuli was enlarged and there was a marked capillary dilation in the peritubular area (Figure 7(b)). The morphology of the renal tubules was also affected as reflected with numerous swollen mitochondria with a crista disarrangement and partial cristolysis within tubular epithelium (Figure 7(c)).

## 6. Discussion

Progressive heart failure is associated with growing deterioration of kidney function which in turn leads to a hemodynamic and neurohumoral worsening of heart failure, thereby, increasing the risk of mortality by 7.5-fold [1]. This study investigated the possible mechanisms that underlie renal damage following heart dysfunction using the rat model of infrarenal aortocaval fistula-induced heart failure. Kidney tissue sections of these animals showed light microscopic alterations such as congestion, glomerular atrophy with widening of Bowman's space, edema with epithelial shedding in tubular structures, pyknotic nuclei, desquamated cells, and hemorrhages suggesting apoptotic events. Consistently, double immunofluorescent staining revealed enhanced expression of proapoptotic Bax, mitochondrial leakage of cytochrome C, and nuclear transfer of activated caspase-3 indicating apoptotic processes. Finally, electron microscopy confirmed the presence of apoptosis by signs of nuclear condensation, DNA fragmentation, shrinkage, and dissolution of the mitochondrial structure. These findings provide morphological evidence of apoptotic events during kidney injury resulting from congestive heart failure.

Recently, our group successfully modified an experimental model of heart failure by the induction of an infrarenal aortocaval fistula which resulted in overt signs of congestive heart failure within a predictable short-time period [17]. Heart performance and kidney function are closely interconnected, and a close link exists between these organs [1]. Dysfunction of one organ often leads to a deterioration of function of the other one such as the kidney [27]. Recently, the investigation of organ crosstalk in acute renal injury reported that cell death, inflammation, cytokine and chemokine overexpression, caspase-mediated apoptotic mechanisms, and oxidative stress might induce distant organ dysfunction [28, 29]. Consistently, the progression of heart failure may contribute to gradual toxic injury to renal cells including apoptosis and consequently persistent kidney damage and functional loss [5, 9]. Therefore, we investigated pathological changes in the kidney following ACF-induced heart failure in rats. Our histological investigation revealed that the glomeruli and tubuli in the kidneys of heart failure rats showed morphological and pathological changes such as mitochondrial condensation and nuclear degeneration suggesting apoptotic cell death as compared to controls. Consistently, kidney apoptosis was observed by TUNEL staining as reflected with TUNEL-positive apoptotic tubular cells in the kidney following ACF-induced heart failure. Our findings are in agreement with a previous study showing that the microvascular endothelial permeability, inflammation, and tubular cell apoptosis significantly increased in rat kidneys following left coronary artery-induced myocardial infarction [11].

Then, we further corroborated evidence for apoptotic events by demonstrating increased expression of the proapoptotic protein Bax in renal tubule epithelial cells and glomeruli after heart failure induction. Our findings are in agreement with the previous report by Yang et al., [30] showing that Bax overexpression positively correlated with caspase-3 activity and subsequent renal apoptosis that was associated with renal inflammation, tubular atrophy, and renal fibrosis in experimental glomerulonephritis. Recent evidence is emerging that the mitochondria-mediated apoptosis is initiated by a variety of apoptosis-inducing signals that cause the imbalance of the major apoptosis regulator such as Bax [15]. Indeed, the previous studies reported that the proapoptotic protein Bax after being activated triggers the disruption of mitochondrial transmembrane potential, followed by mitochondrial swelling and an increase in the permeability of the outer mitochondrial membrane [12–14]. Therefore, we extended our investigation to determine the apoptotic pathway-mediated mitochondrial enzyme cytochrome C in renal cell death during heart failure. Indeed, our immunofluorescence confocal microscopy revealed that cytochrome C immunoreactivity was confined primarily to mitochondria within renal tubule epithelial cells in control rats; however, it leaked into the cytosol of renal tubule epithelial cells following heart failure, where it no longer colocalized with mitochondria. This is in agreement with previous studies reporting that cytochrome C released from mitochondria to the cytosol initiates caspase activation in isolated kidney cortex after cadmium-induced apoptosis [31]. Similarly, another previous study reported that cytochrome C is released from mitochondria to the cytosol during apoptotic events such as hypertensive nephrosclerosis in rats [32].

We further corroborated the evidence for apoptotic pathway-mediated renal cell death by providing further morphological evidence for the activation of caspase-3 from cell organelles like structures that could be shown to translocate in the cleaved form into the perinuclear area of renal tubule epithelial and glomerular cells after heart failure. Our results receive good support from previous studies demonstrating the activation of caspase-3 in cisplatin-induced renal injuries [33] as well as in polymyxin-induced apoptosis in rat kidney proximal tubular cells [34].

In summary, heart failure often leads to the damage of the kidneys, a phenomenon called cardiorenal syndrome [9]. Our present study demonstrates that progressive heart failure [21] is associated with obvious apoptotic events with nucleus degeneration, mitochondrial swelling, and cell death in the glomerular and tubular area of the kidney. In parallel, under normal conditions, cytochrome C colocalized primarily with cell mitochondria and cleaved caspase-3 was absent. In heart failure, the overexpression of proapoptotic Bax protein concomitant with cytochrome C released from the outer mitochondrial membrane into cell cytoplasm led to subsequent caspase-3 activation triggering apoptotic events. These findings provide morphological evidence of renal injury resulting from congestive heart failure and support the pathological interactions between the heart and kidney during heart failure.

## Figures and Tables

**Table 1:** Hemodynamic parameters.

	Control (*n* = 5)	ACF (*n* = 5)
SV (*μ*l)	134 ± 3^∗^	298 ± 40^∗^
LVEF (%)	74 ± 2^∗^	45 ± 4^∗^
LVEDP (mmHg)	5.1 ± 0.3^∗^	12.1±1^∗^
ZVD (mmHg)	0.1 ± 0.1^∗^	5.6 ± 1^∗^
HR (min^−1^)	340.41 ± 10^+^	316.98 ± 10^+^
LVESV (*μ*l)	48.18 ± 5^∗^	359.71 ± 36^∗^
LVEDV (*μ*l)	181.86 ± 8^∗^	658.04 ± 61^∗^

^∗^ *p* < 0.05. + indicates *p* = 0.717.
